# Supplementary material for: Reliability of a Risk-Factor Questionnaire for Osteoporosis: A Primary Care Survey Study with Dual Energy X-ray Absorptiometry Ground Truth
Source: Int J Environ Res Public Health. 2021 Jan 28;18(3):1136. doi: 10.3390/ijerph18031136 (PMC7908374; doi:10.3390/ijerph18031136)
Supplement: Supplementary file 1 [file ijerph-18-01136-s001.zip › Supplement/Figure S1.docx]

## Supplemental Figure S1

## Relibility of a risk-factor questionnaire for osteoporosis: a primary care survey study with Dual Energy X-ray Absorptiometry ground truth

**Authors**

Maria Radeva^1^, Dorothee Predel^2†^, Sven Winzler^3^, Ulf Teichgräber^4^, Alexander Pfeil^5^, Ansgar Malich^6^ and Ismini Papageorgiou^7,8*^

**Affiliations**

^1^Institute of Diagnostic and Interventional Radiology, Jena University Hospital – Friedrich Schiller University Jena, Am Klinikum 1, 07747, Jena, Germany; e-mail: maria.radeva@yahoo.de

^2^Institute of Radiology, Suedharz Hospital Nordhausen, Dr.-Robert-Koch-Str. 39, 99734 Nordhausen, Germany; e-mail: [dteepredel@t-online.de](mailto:dteepredel@t-online.de)

^3^Institute of Radiology, Suedharz Hospital Nordhausen, Dr.-Robert-Koch-Str. 39, 99734 Nordhausen, Germany; e-mail: [sven.winzler@shk.ndh.de](mailto:sven.winzler@shk.ndh.de)

^4^Institute of Diagnostic and Interventional Radiology, Jena University Hospital – Friedrich Schiller University Jena, Am Klinikum 1, 07747, Jena, Germany; e-mail: ulf.teichgraeber@med.uni-jena.de

^5^Department of Internal Medicine III, Jena University Hospital – Friedrich Schiller University Jena, Am Klinikum 1, 07747 Jena, Germany; e-mail: [alexander.pfeil@med.uni-jena.de](mailto:alexander.pfeil@med.uni-jena.de)

^6^Institute of Radiology, Suedharz Hospital Nordhausen, Dr.-Robert-Koch-Str. 39, 99734 Nordhausen, Germany; e-mail: ansgar.malich@shk-ndh.de

^7^Institute of Diagnostic and Interventional Radiology, Jena University Hospital – Friedrich Schiller University Jena, Am Klinikum 1, 07747, Jena, Germany, and

^8^Institute of Radiology, Suedharz Hospital Nordhausen, Dr.-Robert-Koch-Str. 39, 99734 Nordhausen, Germany; e-mail: ismini.papageorgiou@shk.ndh.de

^†^current address: Department for Nuclear Medicine, Central Hospital Bad Berka, Robert-Koch-Allee 9, 99437 Bad Berka, Germany

^*^**Correspondence**: Ismini Papageorgiou M.D.,M.Sc.,Ph.D.; e-mail: Ismini.papageorgiou@shk-ndh.de ORCID: 0000-0001-5810-483





**Fig. S1** Response rate, the age profile of responders and non-responders for each question Q1/Q24. R, responders; Rf, responder females; Rm, responder males; NR, non-responders; NRf, non-responder females; NRm, non-responder males
