# Supplementary material for: Reliability of a Risk-Factor Questionnaire for Osteoporosis: A Primary Care Survey Study with Dual Energy X-ray Absorptiometry Ground Truth
Source: Int J Environ Res Public Health. 2021 Jan 28;18(3):1136. doi: 10.3390/ijerph18031136 (PMC7908374; doi:10.3390/ijerph18031136)
Supplement: Supplementary file 1 [file ijerph-18-01136-s001.zip › Supplement/TableS1.docx.docx]

## Supplemental Table S1

## Relibility of a risk-factor questionnaire for osteoporosis: a primary care survey study with Dual Energy X-ray Absorptiometry ground truth

**Authors**

Maria Radeva^1^, Dorothee Predel^2†^, Sven Winzler^3^, Ulf Teichgräber^4^, Alexander Pfeil^5^, Ansgar Malich^6^ and Ismini Papageorgiou^7,8*^

**Affiliations**

^1^Institute of Diagnostic and Interventional Radiology, Jena University Hospital – Friedrich Schiller University Jena, Am Klinikum 1, 07747, Jena, Germany; e-mail: maria.radeva@yahoo.de

^2^Institute of Radiology, Suedharz Hospital Nordhausen, Dr.-Robert-Koch-Str. 39, 99734 Nordhausen, Germany; e-mail: [dteepredel@t-online.de](mailto:dteepredel@t-online.de)

^3^Institute of Radiology, Suedharz Hospital Nordhausen, Dr.-Robert-Koch-Str. 39, 99734 Nordhausen, Germany; e-mail: [sven.winzler@shk.ndh.de](mailto:sven.winzler@shk.ndh.de)

^4^Institute of Diagnostic and Interventional Radiology, Jena University Hospital – Friedrich Schiller University Jena, Am Klinikum 1, 07747, Jena, Germany; e-mail: ulf.teichgraeber@med.uni-jena.de

^5^Department of Internal Medicine III, Jena University Hospital – Friedrich Schiller University Jena, Am Klinikum 1, 07747 Jena, Germany; e-mail: [alexander.pfeil@med.uni-jena.de](mailto:alexander.pfeil@med.uni-jena.de)

^6^Institute of Radiology, Suedharz Hospital Nordhausen, Dr.-Robert-Koch-Str. 39, 99734 Nordhausen, Germany; e-mail: ansgar.malich@shk-ndh.de

^7^Institute of Diagnostic and Interventional Radiology, Jena University Hospital – Friedrich Schiller University Jena, Am Klinikum 1, 07747, Jena, Germany, and

^8^Institute of Radiology, Suedharz Hospital Nordhausen, Dr.-Robert-Koch-Str. 39, 99734 Nordhausen, Germany; e-mail: ismini.papageorgiou@shk.ndh.de

^†^current address: Department for Nuclear Medicine, Central Hospital Bad Berka, Robert-Koch-Allee 9, 99437 Bad Berka, Germany

^*^**Correspondence**: Ismini Papageorgiou M.D.,M.Sc.,Ph.D.; e-mail: Ismini.papageorgiou@shk-ndh.de ORCID: 0000-0001-5810-483

| **Table S1** Survey questionnaire |  |  |
| --- | --- | --- |
| Please answer if you have any of the below pathologies to the best of your knowledge | **Yes** | **No** |
| Q1 a chronic bowel disease? |  |  |
| Q2 rheumatoid arthritis? |  |  |
| Q3 a malabsorption syndrome? |  |  |
| Q4 a kidney failure or other kidney disease? |  |  |
| Q5 liver insufficiency, or another liver disease? |  |  |
| Q6 an eating disorder (anorexia, bulimia), or are you underweight? |  |  |
| Q7 a growth disorder? |  |  |
| Q8 a developmental delay? |  |  |
| Q9 a premature menopause before the age of 45? |  |  |
| Q10 prolonged menopause? |  |  |
| Q11 an organ transplant? |  |  |
| Q12 proven osteoporosis? |  |  |
| Q13 a bone disease (osteogenesis imperfecta, myelomatosis, fluorosis, rickets, or other)? |  |  |
| Q14 a connective tissue disease (Marfan syndrome or other)? |  |  |
| Q15 Do you regularly take glucocorticoids (steroids, cortisone), or do you suffer from Cushing's disease? |  |  |
| Q16 Do you regularly take anticonvulsant drugs (against epileptic seizures)? |  |  |
| Q17 Has your height decreased by more than 4cm? |  |  |
| Q18 Have you been immobilized by an illness for a long time? |  |  |
| Q19 To the best of your knowledge, have you had a fracture of a bone without an adequate accident mechanism? |  |  |
| Q20 Are you aware of malfunctioning thyroid, parathyroid, or other glands? |  |  |
| Q21 Are you suffering from a lack of sex hormones? |  |  |
| Q22 Do you take heparin or other anticoagulant medication regularly? |  |  |
| Q23 Have you had a malignant disease with appropriate chemotherapy treatment? |  |  |
| Q24 Are you at increased familial risk for osteoporosis? |  |  |
