# Supplementary material for: Reliability of a Risk-Factor Questionnaire for Osteoporosis: A Primary Care Survey Study with Dual Energy X-ray Absorptiometry Ground Truth
Source: Int J Environ Res Public Health. 2021 Jan 28;18(3):1136. doi: 10.3390/ijerph18031136 (PMC7908374; doi:10.3390/ijerph18031136)
Supplement: Supplementary file 1 [file ijerph-18-01136-s001.zip › Supplement/TableS2.docx.docx]

| Table S2 Risk factor correlation with increased osteoporosis incidence | | | | | | | | | | | | | | | | | | | |
| --- | --- | --- | --- | --- | --- | --- | --- | --- | --- | --- | --- | --- | --- | --- | --- | --- | --- | --- | --- |
| N total (male/female) = 553 (78/475), Age (68±12 / 68±13) y.o. | Ground truth hip, index osteoporosis | | | | | | | | | | Ground truth min T score, index osteoporosis | | | | | | | | |
| Survey Question | **Univariate analysis, linear regression (spearman)** | | | | **Multiple logistic regression, Feed all 24 covariates** | | | | | | **Univariate analysis, linear regression (spearman)** | | | **Multiple logistic regression** | | | | | |
|  |  |  |  |  | Hosmer-Lemeshow Statistic ALL: 2.700 (P = 0.952)  Hosmer-Lemeshow Statistic FEM: 0.626 (P = 1.000) | | | | | |  |  |  | Hosmer-Lemeshow Statistic ALL: 9.345 (P = 0.314)  Hosmer-Lemeshow Statistic: 3.484 (P = 0.900) | | | | | |
|  | ***N*** | ***P*** | ***R*** | ***R^2^*** | ***N*** | ***Coefficient*** | ***SE*** | ***Wald statistic, chi-squared*** | ***P*** | ***Odds ratio*** | ***P*** | ***R*** | ***R^2^*** | ***N*** | ***Coefficient*** | ***SE*** | ***Wald statistic chi- squared*** | ***P*** | ***Odds***  ***ratio*** |
| Q1 IBD total | *544* | 0.912 | 0.005 | 0.000 | 211 | 0.8580 | 1.3070 | 0.4310 | 0.5110 | 2.3590 | 0.8400 | 0.0087 | 0.0001 | 211 | -0.1060 | 0.7690 | 0.0191 | 0.8900 | 0.8990 |
| Q1 IBD female | 468 | 0.461 | 0.3420 | 0.0012 | 174 | -36.0090 | >1000 | 0.0000 | 0.9970 | 0.0000 | 0.7420 | 0.0153 | 0.0002 | 174 | -1.3850 | 1.2140 | 1.3010 | 0.2540 | 0.2500 |
| Q2 RA total | 523 | 0.5100 | 0.0288 | 0.0008 |  | -0.6370 | 0.8720 | 0.5340 | 0.4650 | 0.5290 | 0.2550 | 0.4990 | 0.0025 |  | ***-1.0730*** | ***0.5480*** | ***3.8370*** | ***0.0500*** | ***0.3420*** |
| Q2 RA female | 450 | 0.6730 | 0.0199 | 0.0004 |  | -19.4040 | >1000 | 0.0000 | 0.9970 | 0.0000 | 0.5090 | 0.0312 | 0.0010 |  | ***-2.4340*** | ***0.9780*** | ***6.1920*** | ***0.0130*** | ***0.0877*** |
| Q3 MAL total | 485 | 0.8110 | 0.0199 | 0.0001 |  | -16.1840 | >1000 | 0.0000 | 0.9990 | 0.0000 | 0.4890 | 0.0315 | 0.0010 |  | -15.8930 | >1000 | 0.0000 | 0.9960 | 0.0000 |
| Q3 MAL female | 421 | 0.6830 | 0.0199 | 0.0004 |  | 19.0600 | >1000 | 0.0000 | 1.0000 | >1000 | 0.7520 | 0.0154 | 0.0002 |  | -16.2250 | >1000 | 0.0000 | 0.9980 | 0.0000 |
| Q4 RI total | 538 | 0.4870 | 0.0300 | 0.0009 |  | -1.1150 | 1.3500 | 0.6830 | 0.4090 | 0.3280 | 0.1230 | 0.0666 | 0.0026 |  | 0.9070 | 0.7080 | 1.6440 | 0.2000 | 2.4780 |
| Q4 RI female | 463 | 0.3510 | 0.0434 | 0.0019 |  | -42.5400 | >1000 | 0.0000 | 0.9970 | 0.0000 | 0.1850 | 0.0618 | 0.0038 |  | -0.4110 | 1.3490 | 0.0929 | 0.7610 | 0.6630 |
| Q5 LI total | 538 | 0.6620 | 0.0189 | 0.0004 |  | 1.9520 | 1.7470 | 1.2490 | 0.2640 | 7.0440 | 0.1450 | 0.0630 | 0.0040 |  | -0.4640 | 1.1350 | 0.1670 | 0.6830 | 0.6290 |
| Q5 LI female | 462 | 0.6880 | 0.0187 | 0.0004 |  | 25.1130 | >1000 | 0.0000 | 0.9960 | >1000 | 0.1910 | 0.0609 | 0.0037 |  | -0.5750 | 1.6880 | 0.1160 | 0.7330 | 0.5630 |
| Q6 ED total | ***545*** | ***<0.001*** | ***0.1970*** | ***0.0390*** |  | ***5.2650*** | ***1.4960*** | ***12.3810*** | ***<0.001*** | ***193.5130*** | 0.1530 | 0.0163 | 0.0038 |  | ***2.3380*** | ***1.0700*** | ***4.7760*** | ***0.0290*** | ***10.3600*** |
| Q6 ED female | ***469*** | ***<0.001*** | ***0.2190*** | ***0.0479*** |  | ***6.2800*** | ***2.3800*** | ***6.9600*** | ***0.0080*** | ***533.9250*** | 0.0870 | 0.0790 | 0.0062 |  | ***2.9230*** | ***1.2900*** | ***5.1330*** | ***0.0230*** | ***18.5980*** |
| Q7 Growth Ret total | 543 | 0.4490 | 0.0325 | 0.0011 |  | -12.2360 | >1000 | 0.0000 | 0.9990 | 0.0000 | 0.4030 | 0.0359 | 0.0013 |  | -14.8150 | >1000 | 0.0000 | 0.9960 | 0.0000 |
| Q7 Growth Ret female | 469 | 0.5190 | 0.0298 | 0.0009 |  | n.a. | n.a. | n.a. | n.a. | n.a. | 0.7060 | 0.0174 | 0.0003 |  | n.a. | n.a. | n.a. | n.a. | n.a. |
| Q8 Dev Ret total | 537 | 0.5350 | 0.0268 | 0.0007 |  | 53.5350 | >1000 | 0.0000 | 0.9980 | >1000 | 0.7510 | 0.0137 | 0.0002 |  | 10.2280 | >1000 | 0.0000 | 0.9990 | >1000 |
| Q8 Dev Ret female | 465 | 0.5750 | 0.0261 | 0.0007 |  | n.a. | n.a. | n.a. | n.a. | n.a. | 0.9940 | 0.0004 | 0.0000 |  | n.a. | n.a. | n.a. | n.a. | n.a. |
| Q9 PreMen female | 462 | 0.4310 | 0.0367 | 0.0014 |  | -4.7950 | 3.0720 | 2.4360 | 0.1190 | 0.0083 | 0.7870 | 0.0126 | 0.0002 |  | -0.0944 | 0.7100 | 0.0177 | 0.8940 | 0.9100 |
| Q10 ProMen female | 445 | 0.8140 | 0.1120 | 0.0001 |  | 2.6280 | 1.7080 | 2.3660 | 0.1240 | 13.8410 | 0.3700 | 0.0426 | 0.0018 |  | 0.5590 | 0.8920 | 0.3930 | 0.5310 | 1.7490 |
| Q11 Trans total | 524 | 0.3890 | 0.0377 | 0.0014 |  | -13.4830 | >1000 | 0.0000 | 0.9990 | 0.0000 | 0.6420 | 0.0203 | 0.0004 |  | -15.0510 | >1000 | 0.0000 | 0.9960 | 0.0000 |
| Q11 Trans female | 457 | 0.5240 | 0.0299 | 0.0009 |  | 23.8990 | >1000 | 0.0000 | 1.0000 | >1000 | 0.6990 | 0.0181 | 0.0003 |  | -13.5620 | >1000 | 0.0000 | 0.9980 | 0.0000 |
| Q12 OP total | ***504*** | ***<0.001*** | ***0.1620*** | ***0.0264*** |  | ***1.4460*** | ***0.7150*** | ***4.0870*** | ***0.0430*** | ***4.2440*** | ***<0.001*** | ***0.3570*** | ***0.1270*** |  | ***1.9030*** | ***0.4190*** | ***20.6720*** | ***<0.001*** | ***6.7060*** |
| Q12 OP female | ***436*** | ***0.0010*** | ***0.1570*** | ***0.0246*** |  | 1.4200 | 0.9580 | 2.1980 | 0.1380 | 4.1390 | ***<0.001*** | ***0.3550*** | ***0.1260*** |  | ***1.9340*** | ***0.5020*** | ***14.8590*** | ***<0.001*** | ***6.9190*** |
| Q13 BD total | 467 | 0.1030 | 0.0715 | 0.0057 |  | 0.6060 | 0.7340 | 0.6810 | 0.4090 | 1.8330 | ***<0.001*** | ***0.2013*** | ***0.0454*** |  | -0.0888 | 0.4760 | 0.0348 | 0.8520 | 0.9150 |
| Q13 BD female | ***407*** | ***0.0030*** | ***0.0735*** | ***0.0054*** |  | 0.9270 | 1.0290 | 0.8120 | 0.3680 | 2.5270 | ***<0.001*** | ***0.2950*** | ***0.4640*** |  | -0.2090 | 0.6130 | 0.1170 | 0.7330 | 0.8110 |
| Q14 CTD total | 508 | 0.2750 | 0.0485 | 0.0024 |  | -14.8220 | >1000 | 0.0000 | 0.9970 | 0.0000 | 0.2260 | 0.0538 | 0.0029 |  | 0.7930 | 1.1930 | 0.4420 | 0.5060 | 2.2100 |
| Q14 CTD female | 441 | 0.3270 | 0.0468 | 0.0022 |  | -18.0850 | >1000 | 0.0000 | 0.9990 | 0.0000 | 0.5460 | 0.0288 | 0.0008 |  | -15.6190 | >1000 | 0.0000 | 0.9970 | 0.0000 |
| Q15 Cort total | 526 | 0.2210 | 0.0535 | 0.0029 |  | -1.3990 | 1.9670 | 0.5060 | 0.4770 | 0.2470 | 0.2510 | 0.0501 | 0.0025 |  | -0.0715 | 0.7010 | 0.0104 | 0.9190 | 0.9310 |
| Q15 Cort female | 454 | 0.4010 | 0.0395 | 0.0016 |  | -17.5910 | >1000 | 0.0000 | 0.9980 | 0.0000 | 0.4920 | 0.0323 | 0.0010 |  | -1.5450 | 1.1800 | 1.7130 | 0.1910 | 0.2130 |
| Q16 Anticonv total | 529 | 0.2110 | 0.0545 | 0.0030 |  | -15.5070 | >1000 | 0.0000 | 0.9990 | 0.0000 | 0.3490 | 0.0408 | 0.0017 |  | 17.5310 | >1000 | 0.0000 | 0.9960 | >1000 |
| Q16 Anticonv female | 457 | 0.2530 | 0.0536 | 0.0029 |  | 47.2470 | >1000 | 0.0000 | 0.9990 | >1000 | 0.9400 | 0.0035 | 0.0000 |  | 32.5200 | >1000 | 0.0000 | 0.9970 | >1000 |
| Q17 Height total | 525 | 0.4020 | 0.0366 | 0.0013 |  | -0.5180 | 0.6970 | 0.5510 | 0.4580 | 0.5960 | 0.1050 | 0.0709 | 0.0050 |  | -0.2100 | 0.3890 | 0.2910 | 0.5900 | 0.8110 |
| Q17 Height female | 453 | 0.4810 | 0.0332 | 0.0011 |  | -0.5190 | 0.9840 | 0.2780 | 0.5980 | 0.5950 | 0.1500 | 0.0677 | 0.0042 |  | -0.2660 | 0.4870 | 0.2990 | 0.5850 | 0.7660 |
| Q18 Immobil total | 509 | 0.3550 | 0.0411 | 0.0017 |  | -0.4330 | 0.8650 | 0.2500 | 0.6170 | 0.6490 | 0.4510 | 0.0335 | 0.0011 |  | 0.1320 | 0.5110 | 0.0667 | 0.7960 | 1.1410 |
| Q18 Immobil female | 438 | 0.2480 | 0.0553 | 0.0031 |  | -3.9780 | 2.5220 | 2.4890 | 0.1150 | 0.0187 | 0.3570 | 0.0441 | 0.0020 |  | -0.1630 | 0.7100 | 0.0528 | 0.8180 | 0.8500 |
| Q19 Fracture total | 515 | 0.5640 | 0.0255 | 0.0007 |  | 1.0230 | 0.7930 | 1.6630 | 0.1970 | 2.7800 | 0.5000 | 0.0298 | 0.0009 |  | -0.0329 | 0.4570 | 0.0052 | 0.9430 | 0.9680 |
| Q19 Fracture female | 443 | 0.4670 | 0.0346 | 0.0011 |  | 0.5900 | 0.9890 | 0.3560 | 0.5510 | 1.8050 | 0.8680 | 0.0079 | 0.0001 |  | -0.4600 | 0.5840 | 0.6210 | 0.4310 | 0.6310 |
| Q20 ThPTh total | 533 | 0.1990 | 0.0557 | 0.0031 |  | ***1.4490*** | ***0.6220*** | ***5.4330*** | ***0.0200*** | ***4.2580*** | 0.1560 | 0.0615 | 0.0038 |  | 0.0997 | 0.3800 | 0.0688 | 0.7930 | 1.1050 |
| Q20 ThPTh female | 457 | 0.1470 | 0.0680 | 0.0046 |  | 0.7340 | 0.9110 | 0.6490 | 0.4210 | 2.0830 | 0.3490 | 0.0439 | 0.0019 |  | -0.1850 | 0.4660 | 0.1570 | 0.6920 | 0.8310 |
| Q21 SexHorm total | ***477*** | ***0.0420*** | ***0.0930*** | ***0.0085*** |  | ***-3.1020*** | ***1.3640*** | ***5.1720*** | ***0.0230*** | ***0.0450*** | ***0.0210*** | ***0.1060*** | ***0.0112*** |  | ***-1.9950*** | ***0.6810*** | ***8.5820*** | ***0.0030*** | ***0.1360*** |
| Q21 SexHorm female | ***414*** | ***0.0400*** | ***0.1010*** | ***0.0102*** |  | ***-4.3590*** | ***1.9880*** | ***4.8060*** | ***0.0280*** | ***0.0128*** | ***0.0150*** | ***0.1200*** | ***0.0144*** |  | ***-2.1990*** | ***0.8360*** | ***6.9260*** | ***0.0080*** | ***0.1110*** |
| Q22 Anticoag total | 536 | 0.1080 | 0.0695 | 0.0048 |  | ***1.8000*** | ***0.6630*** | ***7.3690*** | ***0.0070*** | ***6.0470*** | 0.9330 | 0.0036 | 0.0000 |  | 0.0989 | 0.4060 | 0.0595 | 0.8070 | 1.1040 |
| Q22 Anticoag female | 462 | 0.1350 | 0.0697 | 0.0049 |  | 1.2960 | 0.8970 | 2.0870 | 0.1490 | 3.6550 | 0.9280 | 0.0042 | 0.0000 |  | -0.2680 | 0.5180 | 0.2670 | 0.6060 | 0.7650 |
| Q23 CA total | 536 | 0.2770 | 0.0470 | 0.0022 |  | ***2.1240*** | ***0.9040*** | ***5.5170*** | ***0.0190*** | ***8.3610*** | 0.3960 | 0.0367 | 0.0014 |  | 0.9810 | 0.5660 | 3.0010 | 0.0830 | 2.6670 |
| Q23 CA female | 462 | 0.3180 | 0.0466 | 0.0022 |  | ***3.3730*** | ***1.2560*** | ***7.2100*** | ***0.0070*** | ***29.1600*** | 0.4590 | 0.0345 | 0.0012 |  | ***1.5260*** | ***0.6440*** | ***5.6150*** | ***0.0180*** | ***4.6020*** |
| Q24 Family total | 505 | 0.2690 | 0.0492 | 0.0024 |  | -0.2070 | 0.6320 | 0.1080 | 0.7430 | 0.8130 | 0.5680 | 0.0254 | 0.0006 |  | 0.0865 | 0.3670 | 0.0556 | 0.8140 | 1.0900 |
| Q24 Family female | 433 | 0.346 | 0.0454 | 0.0021 |  | 0.4350 | 0.8670 | 0.2520 | 0.6160 | 1.5450 | 0.8500 | 0.0091 | 0.0001 |  | 0.3690 | 0.4460 | 0.6820 | 0.4090 | 1.4460 |
